# Impact of the COVID-19 Pandemic on Sedentary Time and Behaviour in Children and Adults: A Systematic Review and Meta-Analysis

**DOI:** 10.3390/ijerph182111286

**Published:** 2021-10-27

**Authors:** Adam Runacres, Kelly A. Mackintosh, Rachel L. Knight, Liba Sheeran, Rhys Thatcher, James Shelley, Melitta A. McNarry

**Affiliations:** 1 Applied Sports, Technology, Exercise and Medicine (A-STEM) Research Centre, Swansea University, Swansea SA1 8EN, UK; 918800@Swansea.ac.uk (A.R.); k.mackintosh@swansea.ac.uk (K.A.M.); 974302@Swansea.ac.uk (R.L.K.); james.shelley@swansea.ac.uk (J.S.); 2 School of Healthcare Sciences, College of Biomedical and Life Sciences, Cardiff University, Cardiff CF24 0AB, UK; sheeranl@cardiff.ac.uk; 3 Biomechanics and Bioengineering Research Centre Versus Arthritis, Cardiff University, Cardiff CF24 0AB, UK; 4 Institute of Biological Environmental and Rural Sciences, Aberystwyth University, Ceredigion SY23 3FL, UK; ryt@aber.ac.uk

**Keywords:** mental health, gender, screen time, older adults, country, lockdown

## Abstract

The aim of this meta-analysis was to quantify the change in sedentary time during the COVID-19 pandemic and its effect on health outcomes in the general population. One thousand six hundred and one articles published after 2019 were retrieved from five databases, of which 64 and 40 were included in the systematic review and meta-analysis, respectively. Studies were grouped according to population: children (<18 years), adults (18–64 years) and older adults (>65 years). Average sedentary time was calculated, with sub-analyses performed by country, behaviour type and health outcomes. Children were most affected, increasing their sedentary time by 159.5 ± 142.6 min day^−1^, followed by adults (+126.9 ± 42.2 min day^−1^) and older adults (+46.9 ± 22.0 min day^−1^). There were no sex differences in any age group. Screen time was the only consistently measured behaviour and accounted for 46.8% and 57.2% of total sedentary time in children and adults, respectively. Increases in sedentary time were negatively correlated with global mental health, depression, anxiety and quality of life, irrespective of age. Whilst lockdown negatively affected all age groups, children were more negatively affected than adults or older adults, highlighting this population as a key intervention target. As lockdowns ease worldwide, strategies should be employed to reduce time spent sedentary. Trial registration: PROSPERO (CRD42020208909).

## 1. Introduction

Sedentary behaviour, defined as any activity in a seated or reclined posture expending ≤1.5 metabolic equivalents (METs, [1]), is suggested to be an independent short- and long-term risk factor for markers of adiposity, cardiovascular disease and type II diabetes mellitus [2,3,4]. Similarly, prolonged bouts of uninterrupted sedentary time across the day are associated with significant health risks [3,5] that have been shown to persist irrespective of physical activity (PA) levels [1,6,7], although this remains contentious [8,9]. Indeed, even 10 min of uninterrupted sedentary time has been reported to decrease insulin sensitivity and glucose tolerance and increase circulating triglyceride levels [1]. Of concern, however, the average North American child (8–18 years) engages in 75 h week^−1^ (or 10.7 h day^−1^) of multimedia use [10,11], with comparable levels reported in European [12] and Asian [13] children. Similar levels of sedentary time (up to 12.3 ± 1.4 h day^−1^) have been reported globally in adults [3,4]. Sedentary lifestyles, and the identification of strategies to reverse them, represent a significant public health challenge [14]. Moreover, the need to understand sedentary time and behaviour, and their relationship(s) with health outcomes, may be more important than ever with the emergence of novel Coronavirus 2019 disease (COVID-19).

Since the pandemic was announced in December 2019, there have been in excess of 167 million cases and 3.5 million deaths across 216 countries, as of 24 May 2021 [15]. Whilst the global response has been far from homogeneous most countries have adopted some form of social distancing, homestay (or lockdown) requirements, self-isolation or quarantine measures to limit COVID-19 transmission, and relieve pressure on health care services [16]. Indeed, in April 2020, over 50% of the global population were subject to some form of government restrictions [15], many of which may have had unintended deleterious health consequences. More specifically, homestay strategies may have increased sitting and screen time, due to children participating in online learning and adults working from home [16], whilst decreasing opportunities to break-up prolonged periods of sedentary time/behaviour. The effects of COVID-19 and subsequent restrictions on habitual PA levels have received substantial attention, with a recent review reporting decreased levels of PA globally which was attributed to social distancing measures [17]. However, no review to date has considered the impact of these restrictions on sedentary time, or the associated behaviours.

Meyer et al. [18] reported that ≥8 h day^−1^ of screen time was associated with a greater likelihood of symptoms of depression, loneliness, and stress during the COVID-19 pandemic in 3052 American adults. However, in the same study, total sitting time was not associated with any mental health outcome [18], in accord with Ugboule et al. [19] who reported no associations between sedentary time and emotional well-being in 9142 similarly aged adults. Studies examining the effect of COVID-19 restrictions on sedentary time in children are also conflicting. Indeed, Kang et al. [20] reported trivial associations between sedentary time and mental health outcomes in 4898 Chinese adolescents (16.3 ± 1.3 years), whereas Lu et al. [21] reported a positive association between sedentary time and risk of insomnia, depression, and anxiety in 965 Chinese adolescents (15.3 ± 0.5 years). Moreover, it is pertinent to note the potential for geographically specific variations in this relationship between sedentary time and health outcomes, thereby limiting the conclusions that can be drawn on the basis of single-country analyses. Therefore, a review is urgently needed to consolidate our understanding of COVID-19′s impact on sedentary time/behaviour and associated health outcomes and thereby highlight avenues for future interventions and research.

Therefore, the aim of this review was to assess the influence of COVID-19 and associated government restrictions on sedentary time and/or behaviour and physical, mental, and social health outcomes in the general population. A secondary aim was to examine the strength of association between sedentary time and/or behaviour and health outcomes during the COVID-19 pandemic.

## 2. Methods

### 2.1. Search Strategy and Inclusion Criteria

This systematic review protocol was registered on PROSPERO (registration number: CRD42020208909) and conducted in line with the Preferred Reporting Items for Systematic Reviews and Meta-Analyses (PRISMA) guidelines [22,23]. An online search was conducted on 12 January 2021 across the following databases: EBSCOhost, Medline, SPORTDiscus, Scopus, and Web of Science Core Collection. A date limit was set as 2020–2021 to ensure that only articles concerning COVID-19 were collected. Across these databases, the key search terms were modified to individual requirements, with Boolean and MeSH terms used to search the following terms and their variations: “sedentary time”, “sedentary behaviour”, “screen time”, “sitting time”, “sedentary posture” AND “COVID-19”, “SARS-CoV”, “2019 corona-virus”. The full search terms can be found in the online supplementary material. Two authors (AR and RK) initially screened all abstracts independently before reviewing full-text articles for inclusion. In cases where disagreements regarding the inclusion of a study were unable to be resolved, LS was consulted to reach a consensus, which occurred on three occasions. Of the three articles where there were disagreements, one was included in the final review.

**Figure 1:**
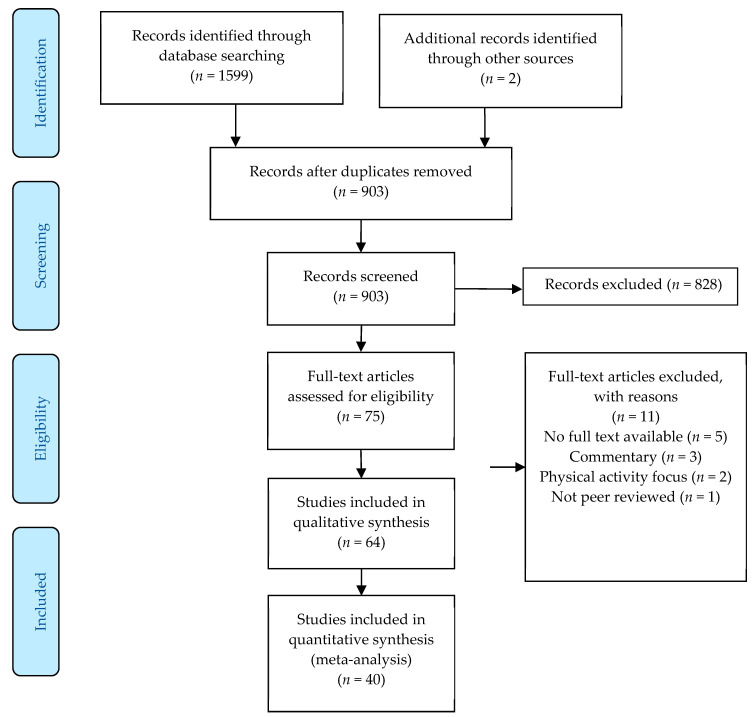
Schematic flow diagram of the systematic review and meta-analysis process.

All studies assessing any type of sedentary behaviour or sedentary time (time spent below 1.5 METs but without verification of posture) using either subjective (questionnaires or interviews) or device-based (accelerometers or inclinometers) measures, in any population (children: <18 years of age, adults: >18 years of age, or older adults: >65 years of age), irrespective of methodological approach (cross-sectional, cohort, or longitudinal study designs), were included. Furthermore, adequate reporting of the restrictions in place at the time of data collection was required for inclusion to enable tentative comparisons to be made between geographical regions. All studies involving human participants and written in the English language which met the specified criteria were included, with any non-peer reviewed grey literature, including conference papers and theses, excluded. To be included within the meta-analysis studies had to provide data from before and during the COVID-19 pandemic so the impact of the pandemic could be assessed. Moreover, commentaries examining the potential detrimental effects of sedentary time and/or behaviour during the COVID-19 pandemic were also excluded. The initial search gathered 1601 results (903 after the removal of duplicates), from which 828 were excluded during the title and abstract screening phase. Following full-text review, 64 articles were retained and included within the systematic review and 40 were utilized within the meta-analysis (Figure 1).

### 2.2. Data Extraction, Quality Assessment, and Risk of Bias Appraisal

Data extraction from all included full-text articles was completed to obtain the following information: authors and year of publication, number and age of participants, country of study, COVID-19 restrictions in place at the time of data collection, sedentary time/behaviour measures, health outcome measures reported (if any), information required to assess risk of bias, and main results (Table 1 and Table 2). All researchers contributed to the synthesis of data. Risk of bias was assessed using the Cochrane risk of bias tool as described in the Cochrane Handbook 5.1.0 for systematic reviews [24]. Briefly, for every included study, risk of bias was classified as ‘High’, ‘Moderate’, ‘Low’ or ‘Unclear/Not reported’ in the following areas: detection bias, attrition bias, selection bias, performance bias, selective reporting bias and other sources of bias. A brief rationale for the decision is included in the supplementary material, in line with previous studies [25,26], with the full risk of bias table available in Supplementary Information Table S1 and Table S2.

The quality of evidence for each of the included studies was decided in a systematic manner with the aid of the Grading of Recommendations Assessment Development and Evaluation (GRADE) framework [27], as used in previous reviews of this type [25,26]. The GRADE framework allows for the classification of research articles into four distinct categories (‘High’, ‘Moderate’, ‘Low’, and ‘Very Low’). Any study including a randomised recruitment and/or a longitudinal design starts at ‘High’, with all other study types and designs starting at ‘Low’. The quality of evidence was then downgraded if there were serious limitations hindering the interpretation of the study, such as high risk of bias, unvalidated data collection methods, and convenience sampling techniques [27]. Conversely, the quality of evidence was upgraded if there is no cause for the downgrading of the study and a large effect size and/or a dose–response relationship was evident within the study [27]. All included studies were synthesised in a systematic approach examining the effect of COVID-19 on overall sedentary time, and specific sedentary behaviours where appropriate. All results were divided into children, adult, and older adult specific segments due to the population-specific sedentary behaviour guidelines [14,28] and the potentially different impacts of government restrictions on these populations.

All meta-analyses were conducted in R (R Studio v1.2.2019, R Studio, Boston, MA, USA) using the meta and metagen packages and their dependencies. Where pre- and post-COVID data were available, the standardised mean difference (SMD) was calculated using the formula: difference between means/standard deviation of outcome [29]. Prior to calculating the SMD, all sedentary time and behaviour measurements were converted into minutes per day (mins day^−1^) to ensure all variables were reported on the same nominal scale. Time spent sedentary during COVID-19 was determined based on those studies that specifically reported the sedentary time of participants experiencing government restrictions and the post data in longitudinal studies. Subsequently, the average sedentary time between studies, and the pooled standard deviation, were calculated. Meta-analyses were conducted separately for both child, adult, and older adult data, and between sexes where the data were available, so that tentative inter-population comparisons could be made.

## 3. Results

There were 64 studies included within the review [18,20,21,27,28,29,30,31,32,33,34,35,36,37,38,39,40,41,42,43,44,45,46,47,48,49,50,51,52,53,54,55,56,57,58,59,60,61,62,63,64,65,66,67,68,69,70,71,72,73,74,75,76,77,78,79,80,81,82,83,84], of which 19 studies included children with 5, 11, and 3 studies scoring high, moderate, and low quality, respectively, on the GRADE scale (Table S1). The remaining 45 studies were conducted with adults (2 in older adults) with 13, 28, and 4 studies scoring high, moderate, and low quality, respectively, on the GRADE scale (Table S2). The 64 studies included within this systematic review encompassed 282,202 participants (28.5 ± 17.1 years), of which 262,630 were adults (93.1%; 36.5 ± 5.5 years), 16,214 were children (5.7%; 11.5 ± 2.3 years) and 3358 older adults (1.2%; 60.6 ± 8.0 years). The majority of the studies were quantitative utilising online questionnaires (60/64; 93.8%), were observational or cross-sectional in design (61/64; 95.2%), had >100 participants (56/64; 87.5%), and recruited from the general population (53/64; 89.0%). Specific populations included elite athletes [43], post-bariatric patients [44], pregnant women [45], participants with depression [82], and children with autism [27], attention deficit hyperactivity disorder (ADHD, [28]), and obesity [29]. Finally, 25 studies (37.5%) were conducted in European countries (Austria, France, Germany, Italy, Poland, Scotland, Spain, and the United Kingdom; [27,29,30,31,32,42,43,45,47,48,49,50,51,52,53,54,55,56,57,58,59,60,61,62]), 18 (28.1%) in Asia (Australia, Bangladesh, China, India, Jordan, Kuwait, Turkey and the United Arab Emirates; [20,21,28,33,34,35,63,64,65,66,67,68,69,70,71,72,73,74]), 12 (18.8%) in North America (Canada and United States; [18,36,37,38,39,40,41,75,76,77,78,79]), 8 (11.0%) in South America (Brazil and Chile; [44,46,80,81,82,83,85,86]), and 1 (1.6%) in Africa (Ghana; [84]).

Overall, participants increased their sedentary time during the COVID-19 pandemic by 135.0 ± 46.0 min day^−1^, however there was a significant difference between children and adults (Table 1 and Table 2, respectively), with children increasing their sedentary time more than adults (+159.5 ± 142.6 min day^−1^ vs. +126.9 ± 42.2 min day^−1^, *p* < 0.05). Only two studies investigated changes in sedentary time in older adults, reporting a non-significant increase of 46.9 ± 22.0 min day^−1^ (Table 2). These increases were found regardless of restrictions currently in place at the time of data collection. Such increases in sedentary time resulted in children, adults and older adults spending 383.9 ± 138.2 min day^−1^, 510.5 ± 167.9 min day^−1^ and 586.3 ± 25.2 min day^−1^ being sedentary, respectively. Despite differences in sedentary time by age, there were no significant differences in sedentary time by sex in children (boys: 367.2 ± 117.6 min day^−1^ vs. girls: 379.5 ± 114.4 min day^−1^) or adults (male: 520.1 ± 181.4 min day^−1^ vs. female: 514.1 ± 163.5 min day^−1^). Breakdowns of total sedentary time by country during the COVID-19 pandemic for children and adults are displayed in Table 3 and Table 4. Cautiously, geographical variations in sedentary time during COVID-19 are apparent in adults, with adults residing in Asian countries engaging in less sedentary time (350.7 ± 184.2 min day^−1^) compared to European (512.2 ± 225.3 min day^−1^), North American (515.0 ± 146.0 min day^−1^), and South American (530.0 ± 20.0 min day^−1^) adults.

Data on specific sedentary behaviours was sparse, with the exception of daily screen time which was suggested to account for 57.2% of total sedentary time (i.e., 274.0 ± 90.1 min day^−1^) in adults. Similarly, screen time was the only specific sedentary behaviour examined consistently in children, accounting for 205.4 ± 23.2 min day^−1^, or 46.8% of total sedentary time. Of the studies that reported associations between changes in sedentary time and health outcomes, the most commonly measured health outcomes were quality of life [20,45,48,61,62,64,68,71,84,85], anxiety and depression [18,20,21,37,46,82] and global mental health [20,42,48,61,62,84,85], with one study measuring the likelihood of seizures in epileptic and otherwise healthy children [32]. The evidence suggests that increases in sedentary time resulting from the COVID-19 restrictions were weakly, but significantly, negatively correlated with quality of life (r^2^ = −0.05, *p* > 0.05; [20,45,48,61,62,64,68,71,84,85]), and global mental health (r^2^ = −0.10, *p* > 0.05; [20,42,48,61,62,84,85]) however it should be noted that not all studies reported significant associations [20]. Conversely, those with a greater sedentary time had a higher likelihood of depression and anxiety (odds ratio: 1.35–1.57; [18,20,21,37,46,82]), although this may depend on the type of sedentary behaviour engaged in [18]. Total daily screen time was significantly associated with the likelihood of a seizure in children, independent of prior seizure history (r^2^ = 0.52–0.57, *p* < 0.01; [32]).

## 4. Discussion

The aim of this meta-analysis was to explore the effects of the COVID-19 pandemic and the associated government restrictions on sedentary time and sedentary behaviour. A key finding was that sedentary time was substantially increased, irrespective of the restrictions imposed or population. More specifically, children increased their sedentary time the most during the pandemic, although their overall time spent sedentary was still lower than observed in adults. There were no significant sex differences in sedentary time across the pandemic irrespective of age. Finally, increases in sedentary time as a result of the pandemic were weakly, but significantly, related with QoL and mental health, with higher time spent sedentary associated with greater anxiety and depression.

### 4.1. Children’s Sedentary Time

The currently available evidence suggests that children increased their overall sedentary time by 159.5 min day^−1^ during the COVID-19 pandemic, although it is pertinent to note the large standard deviation (±142.6 min day^−1^). This therefore highlights the huge disparity in response in this population. Indeed, these results suggest that some children may have accrued similar sedentary time on weekdays during restrictions to during their normal school routines. Whilst it could be postulated that those exhibiting smaller absolute increases in sedentary time may have been characterised by a lower baseline time spent being sedentary and thus the relative increase was still similar, it is of concern to note that some children may have increased their sedentary time by more than five hours per day. Potential reasons for this large discrepancy in sedentary time may be disparities in access to gardens and green spaces during confinement, limiting opportunities to break-up prolonged sedentary time [31,87,88]. More specifically, Akpinar [89] reported that the distance between urban green spaces and the home was significantly correlated with children’s screen time, even after covarying for age and socioeconomic status [SES; [87,90]. Whilst the determinants of sedentary time and behaviour are multifaceted, SES is a critically important factor determining PA and sedentary time in children [90], with COVID-19 exacerbating pre-existing inequalities. Furthermore, other social factors, such as a low parental education level, children with overweight/obese parents [31], and children of parents with high anxiety regarding COVID-19 [37], were all correlates of an increased sedentary time. The finding that screen time accounted for ~47% of total sedentary time was expected given that the majority of countries worldwide have now adopted online learning methods [52].

Despite the apparent increase in sedentary time during the pandemic in children observed in the present meta-analysis, the total sedentary time determined in this review (383.9 ± 138.2 min day^−1^) was nonetheless in accord with [91,92], or lower than [13,93], the figures reported prior to the pandemic. This is likely to be predominantly due to a reliance on subjective recall measures during the pandemic [94]. More specifically, the reliability of recall measures, especially in children, is limited, with boys typically overestimating the amount of PA and sedentary time they perform [95]. Furthermore, children’s misrepresentation of time potentially exaggerates recall bias [95], and that the volume of sedentary time is only one component of a wider composition of daily movement behaviours which are critical for current and future health outcomes [1,91,96,97]. It is also noteworthy that the meta-analysis in children was conducted on only six studies that provided pre-post data, accounting for only 15.0% of the total review population [27,29,30,31,32,38]. Caution must therefore be taken when extrapolating the findings from this meta-analysis to the wider population.

Despite the wide discrepancy in both children’s absolute and increases in sedentary time, there were no sex differences in cumulative sedentary time, which may be considered to be unsurprising given that both boys and girls have been equally affected by lockdown measures. However, pre-COVID-19, it was consistently reported that girls were more sedentary than similarly aged boys [12,93,98,99]. This may therefore indicate that the pandemic had a greater impact on sedentary time in boys than girls. Indeed, despite accruing less overall sedentary time than girls, boys consistently reported an increased screen time [100], with lockdown restrictions likely exaggerating these observations which may explain the lack of sex difference. It is also pertinent to note that previous research has consistently reported that boys are more active during school hours than girls, indicating the social element of PA may be of greater importance to boys [101]. Consequently, the social confinement associated with COVID-19 restrictions may have had a greater impact on boy’s PA levels and subsequent sedentary time. The lack of sex difference may be attributable to different data collection methods between studies, and differences in questions asked, which have varying degrees of reliability [102].

Three studies examined the association of sedentary time with health outcomes in children with conflicting results [20,21,32]. More specifically, Kang et al. [20] reported no association between total sedentary time and perceived mood in 4898 Chinese adolescents (16.3 ± 1.3 years), whereas Lu et al. [21] reported those with a sitting time of ≥4 h day^−1^ had a greater risk of experiencing anxiety, depression and insomnia (OR: 1.35–1.87) in a similar sample of 953 Chinese adolescents (15.3 ± 0.5 years). These differences cannot be attributed to cultural, age or lockdown restrictions and may therefore be indicative of the protective role that the maintenance of PA has on mental health during times of adversity, although more data is needed to confirm this postulation. However, the discrepancy may also be due to differences in the measures of sedentary time used, with Kang et al. [20] reporting total sedentary time while Lu et al. [21] focused specifically on sitting time. Given that the influence of being sedentary on health outcomes is well evidenced to be dependent on specific type of sedentary behaviour [5,26,92,103], further inter-study comparisons are therefore precluded.

### 4.2. Adults and Older Adults

Overall, adults increased their sedentary time by 126.9 ± 42.2 min day^−1^, spending a cumulative 510.5 ± 167.9 min day^−1^ in sedentary behaviours, which was significantly higher than children (+ 127 min day^−1^). The reasons for this are not immediately clear and are likely multifaceted. One possible reason for the higher absolute sedentary time in adults compared to children could be due to their higher sedentary time pre-COVID possibly due to sedentary occupations [104]. Moreover, adults who adhered strictly to guidelines experienced an additional increase of 60 min day^−1^ in sedentary time during the pandemic than those who did not [44]. Furthermore, adults with higher levels of COVID-19 related anxiety were more likely to demonstrate a higher sedentary time compared to those with low anxiety [37]. Additionally, the lack of commute to work due to lockdown and homeworking requirements in most countries and the closure of sporting facilities (gyms, swimming pools etc) is likely to reduce PA opportunities to a greater extent in adults than children. Indeed, it is well accepted that adults complete PA in a structured, planned manner [8,105] and therefore COVID-19 is likely to have a bigger impact upon their PA and sedentary time levels. Taken together, this suggests that children and adults may experience different effects stemming from COVID-19 restrictions and therefore a one-size fits all approach to minimise the health detriments is unlikely to be effective for both populations. Similar to children, there were no sex differences in total sedentary time in adults suggesting men have been disproportionately affected by the effects of COVID-19 restrictions compared to women. Potential reasons for the lack of sex differences include women choosing to partake in physical activity closer to the home [106] which may have been less impacted and the greater proportion of men who regularly partake in team sport activities [107] which were suspended during the pandemic.

Older adults were the most sedentary group included within this systematic review and meta-analysis, with sedentary time totalling 586.3 ± 25.2 min day^−1^. However, they were also the least impacted by COVID-19, with their sedentary time only increasing by an average of 46.9 ± 22.0 min day^−1^. This may be due to their reduced likelihood of being in employment and lower tendency to be highly active prior to COVID-19 [107]. Similar to children, there were no significant sex differences in overall sedentary time, despite females engaging in more sedentary time pre-COVID-19 [4,6]. This may therefore similarly suggest that COVID-19 may have had more of an influence on males’ sedentary time than females’ but more evidence is needed to confirm this postulation.

The regional trends in this meta-analysis were discordant with previous research which reported that Asian adults were the most sedentary [108] and potentially indicate the effect of different lockdown strategies on sedentary time and behaviour. More specifically, China enforced strict, localised lockdowns in areas of infection, and lesser restrictions in areas of lower infection rates, as opposed to blanket national restrictions [109]. Whilst it could be speculated that including participants from non-lockdown regions potentially adds bias within the data, it also provides insights as to how COVID-19 may be managed whilst minimising the undesirable health consequences of lockdown conditions. However, it must be noted that there are a multitude of other factors that influence sedentary time and behaviour [5,110], therefore examining the specific influence of different governmental restrictions globally remains challenging.

Twelve studies examined the effect of sedentary time and behaviour on health-related outcomes in adults [18,45,46,48,61,62,64,68,71,82,84,85]. More specifically, five considered global mental health [18,48,61,84,85] and generally reported a negative association between sedentary time and global mental health, concordant with previous research [111]. However, Stieger et al. [62] reported that sedentary time was positively associated with perceived levels of stress but not overall mental health score, and therefore other lifestyle factors may also be important for the maintenance of mental health during lockdown. Other lifestyle factors noted within the current review include diet [45,51,71,79], physical activity [59,111] and minimising tobacco and alcohol consumption [48,58,79]. Similar relationships were observed regarding the effect of sedentary time on quality of life, [58,62,65,87], depression symptoms [33,82], and subjective well-being [52], highlighting the significant, and diverse, negative consequences of excessive sedentary time. Strategies need to be employed to encourage the breaking up of prolonged sedentary periods and the re-opening of sports facilities and green spaces needs to be prioritised when restrictions are eased for general health and well-being.

### 4.3. Strengths and Limitations

Whilst there are many strengths of this review, including the meta-analysis to quantify changes in sedentary time during the COVID-19 pandemic, the identification of inter-country differences, and the inclusion of both children and adults, there are some limitations which must be acknowledged. First, the global pandemic is still ongoing, with scientific knowledge in this area being updated weekly, which may impact the overall findings. Nevertheless, this review consolidates our current understanding and highlights areas which require further research. Secondly, the majority of studies did not comprehensively report the lockdown restrictions currently in place during the time of data collection, using phrases such as ‘lockdown’ or ‘home-stay’ requirements which were interpreted differently in almost every country [15]. Therefore, no evidence is available to assess the impact of specific lockdown restrictions on sedentary time and behaviours. Additionally, little information was able to be synthesised within this review on the effects of specific sedentary behaviours which are purported to have differing health effects [3,4,58]. Moreover, the reliance on subjective recall data, especially in children, has recognised limitations and questionable validity [102] and therefore the results of this meta-analysis should be interpreted as an estimation only regarding the effect of COVID-19 on sedentary time in children, adults, and older adults. Finally, SES, a strong correlate of sedentary time, was not able to be controlled for within this meta-analysis due to a paucity of available data and needs considering in future work.

## 5. Conclusions

In conclusion, the COVID-19 pandemic resulted in increased sedentary time irrespective of lockdown conditions or population. Of importance, greater increases in time spent sedentary were suggested to be evident in both boys and men, suggesting that they have been disproportionally affected by lockdown restrictions. Moreover, increases in sedentary time as a result of COVID-19 restrictions were weakly, but significantly, correlated to poorer QoL, global mental health, depression, and likelihood of seizures. Therefore, as restrictions ease there should be a focus on reengaging everybody with PA and encouraging the breaking up of prolonged sedentary periods to improve both physical and mental well-being.

## Figures and Tables

**Table 1:** Data extraction of studies measuring sedentary time and/or behaviour during COVID-19 in children.

Author	Population	Country	Restrictions	Sedentary Behaviour Measurement	Health Outcome Measures	Key Findings
Kang et al. [20]	4898 adolescents 16.3 ± 1.3 years	China	School closures and social distancing	IPAQ-Short Form	Mood (Anger, Tension, Fatigue, Depression, Confusion, Self-esteem and Vigor)	Sedentary time totaled 363.6 ± 148.4 min day^−1^ No sig. correlation between sedentary time and any mood during COVID restrictions
Lu et al. [21]	965 adolescents 15.3 ± 0.5 years	China	Social distancing and lockdown/homestay requirements	IPAQ-Short Form	Mental Health outcomes including Insomnia, Depression and Anxiety	54% adolescents sedentary for ≥ 4 h day^−1^ Sedentary time associated with higher odds of experiencing insomnia (OR: 1.60), depression (OR: 1.57) and anxiety (OR: 1.35) during lockdown
Garcia et al. [27]	11 adolescents with Autism 16.9 ± 1.4 years	Florida, USA	Lockdown/Homestay requirements	IPAQ Short Form	-	Number of days where participants met the PA guidelines ↓ from 4.2 ± 1.5 days to 2.3 ± 2.2 days Screen time ↑ by 2.6 h⋅day^−1^ during the week and +1.4 h⋅day^−1^ on the weekend
Sciberras et al. [28]	213 children with ADHD 11.0 ± 3.9 years	Australia	Social distancing and lockdown/homestay requirements	CoRonavIruS Health Impact Survey (CRISIS)	-	Number of children reporting watching TV, social media, and gaming for >1 h⋅day^−1^ increased by 10.2%, 10.8% and 16.9%, respectively
Pietrobelli et al. [29]	44 obese children 13.0 ± 3.1 years	Italy	Lockdown/homestay requirements	Two different interview techniques		Screen time ↑ by 4.9 ± 2.4 h⋅day^−1^ during the lockdown to 7.6 ± 2.1 h⋅day^−1^
Lopez-Beuno et al. [30]	860 children 9.6 ± 3.9 years	Spain	Social distancing, quarantine, and lockdown/homestay requirements	Adapted pre-existing questionnaires	-	Daily screen time ↑ 2.9 ± 2.3 h⋅day^−1^ No significant sex differences in screen time
Medrano et al. [31]	291 children (113 longitudinal) 12.1 ± 2.6 years	Spain	Social distancing and lockdown/homestay requirements	Youth PA (YPA) questionnaire	-	Screen time ↑ 1.9 ± 2.6 h day^−1^ to 6.4 ± 2.4 h day^−1^ Screen time higher in families of non-Spanish origin and lower education level
Palladino et al. [32]	57 children 8.0 ± 1.6 years	Italy	Lockdown/stay at home orders	Questionnaires to assess total screen time	Likelihood of having a seizure	Daily screen time ↑ from 2.5–5.8 h day^−1^ from pre-COVID to circa-COVID Sig. correlation between screen time and seizures for epileptic patients (*r*^2^ = 0.52) and those with no prior history of seizures (*r*^2^ = 0.57)
Dutta et al. [33]	153 participants No overall demographics reported	India	Lockdown/homestay requirements	Parenting practice scale (PPS)	-	Number of youth reporting using phones, watching TV, Laptops and Tablets from 4–8 h⋅day^−1^ increased by 8.7%, 5.7%, 2.8% and 0%, respectively
Eyimaya and Irmak [34]	1115 participants 9.0 ± 2.0 years	Turkey	Lockdown/homestay requirements	IPAQ long-form		71.7% parents reported an ↑ screen time of approximately 6.4 ± 3.0 h⋅day^−1^
Munasinghe et al. [35]	582 adolescents 17.0 ± 1.0 years	Australia	Social distancing, school closures and lockdown/homestay requirements	PACE + Adolescent Physical Activity Measures	-	41.5% were on social media for ≥ 4 h day^−1^ 55.1% watched TV for ≥ 1 h⋅day^−1^
Carroll et al. [36]	310 participants 5.7 ± 2.0 years	Canada	School closures, closure of parks, and social distancing	IPAQ Short Form	-	Screen time = 2.4 ± 1.6 h⋅day^−1^
McCormack et al. [37]	345 parents 10.8 ± 4.0 years	Canada	Social distancing and quarantine measures	Parental recall of child’s PA and sedentary time	Parental COVID anxiety	Majority of children spent ≥ 2 h day^−1^ watching TV (74.1%), using a computer (63.7%), and using screen-based devices (60.7%) Children of highly anxious parents more likely to be sedentary (OR: 1.78; 1.02–3.11)
Schmidt et al. [38]	1174 children No overall demographics reported	Germany	Social distancing and lockdown/homestay requirements	Subjective recall questions	-	4–5 year olds screen time ↑ 41.1 min day^−1^ 6–10 year olds screen time ↑ 67.5 min day^−1^ 11–13 year olds screen time ↑ 60.7 min day^−1^ 14–17 year olds screen time ↑ 67.8 min day^−1^
Dunton et al. [39]	211 children 8.7 ± 2.6 years	United States	Closure of schools, parks, and sports cancelled Social Distancing	Recall questionnaires used to estimate screen time	-	Boys TV time 95.7 ± 68.7 min day^−1^ vs. girls 113.0 ± 79.6 min day^−1^ Media time for leisure use: boys 30.2 ± 53.4 min day^−1^ vs. girls 46.4 ± 68.0 min day^−1^ Children engaged in > 8 h⋅day^−1^ in leisure-related sitting
Mitra et al. [40]	1472 participants No overall demographics reported	Canada	Social distancing and lockdown/homestay requirements	Online questionnaires—limited details on specifics	-	78.8% of children reported an increase in screen time 44.6% reported an increase in social media use 48.6% reported an increase in ‘non-screen based’ sedentary behaviour
Moore et al. [41]	1472 children and adolescents Children: 8.1 ± 2.0 years Youth: 14.9 ± 1.7 years	Canada	Social distancing and lockdown/homestay requirements	participACTION survey	-	Screen time (children)—4.1 h day^−1^ Screen time (youth)—5.0 h day^−1^
Francisco et al. [42]	1480 children 9.1 ± 4.3 years	Multinational (Italy, Spain and Portugal)	Social distancing and lockdown/homestay requirements	IPAQ and the adult sedentary behaviour questionnaire (ASBQ)	-	Number of children reported to engage in >180 min⋅day^−1^ of screen time ↑ 26.6%

ADHD = Attention Deficit Hyperactivity Disorder, CI = Confidence Intervals, COVID-19 = novel coronavirus disease 2019, IPAQ = International Physical Activity Questionnaire, OR = Odds Ratio, PA = Physical Activity, SB = Sedentary Behaviour, ST = Sedentary Time, TV = Television.

**Table 2:** Data extraction of studies measuring sedentary time and/or behaviour during COVID-19 in adults and older adults.

Author	Population	Country	Restrictions	Sedentary Behaviour Measurement	Health Outcome Measures	Key Findings
Meyer et al. [18]	3052 participants No overall descriptives available	United States	Social distancing, quarantine, and lockdown/homestay requirements	Online questionnaires—no details on specific ones used	Depressive, anxiety, loneliness, and stress symptoms Social network and positive mental health all assessed	Those who maintained a screen time of <8 h day^−1^ had sig less depressive symptoms (*b* = 1.9, *p* < 0.01), loneliness (*b* = 0.3, *p* < 0.01), stress (*b* = 0.6, *p* < 0.01) and had a more positive outlook (*b* = 0.92, *p* < 0.01) Sitting time was not associated with any mental health outcome
Carroll et al. [36]	351 participants 38.5 ± 5.2 years	Canada	School closures, closure of parks, and social distancing	IPAQ Short Form	-	Adult sitting time 6.3 ± 3.0 h day^−1^ Screen time 2.8 ± 1.7 h day^−1^
Zinner et al. [43]	14 professional kayakers 22.9 ± 1.4 years	Germany	Social distancing and Lockdown/homestay requirements	Heart rate monitoring	-	Sitting time ↑ from 623.0 ± 63.0 min day^−1^ to 729 ± 21 min day^−1^ during COVID)
Rezende et al. [44]	37 post-bariatric patients 48.1 ± 4.0 years	Brazil	Social distancing and lockdown/homestay requirements	GT3X accelerometers and subjective recall questionnaires	-	Mean sedentary time was 9.5 ± 0.5 h day^−1^ Participants who adhered to social distancing measures spent more time sedentary (+1.1 ± 1.0 h day^−1^)
Bivia-Roig et al. [45]	90 pregnant women 33.1 ± 4.6 years	Spain	Lockdown/homestay requirements	Adapted questionnaires for SB and EuroQol-5D for mental health	Health-Related QoL	↓ in HRQoL 50% ↑ in sitting time (4 h day^−1^–8 h day^−1^)
Werneck et al. [46]	43,995 participants 43.0 ± 0.5 years	Brazil	Social distancing and quarantine measures	No specific details on questionnaires used	Depression	↑ TV viewing time associated with ↓ mental health
Castaneda-Babarro et al. [47]	3800 participants 42.7 ± 10.4	Spain	Lockdown/homestay requirements	Internally validated questionnaire	-	Overall sitting time ↑ 23.8% to 480.0 ± 306.0 min day^−1^ Women less of an increase in sedentary time than men (↑ 25.3% and 35.0%, resp.)
Cheval et al. [48]	110 participants No overall demographics available	France and Switzerland	Limit to 1 h per day exercise Social distancing Home working	Newly designed questionnaire	Global physical and mental health Depressive symptoms Subjective vitality	↑ 75 min day^−1^ sedentary time ↑ sedentary time led to ↓ physical and mental health and subjective vitality
Colivicchi et al. [49]	124 participants 71.0 ± 14.0 years	France	Lockdown/homestay requirements	Telephone interviews	-	41.9% reported ↓ physical activity 50% reported ↑ screen time
Gallé et al. [50]	1430 participants 22.9 ± 3.5 years	Italy	Lockdown/homestay requirements	PLifeCOVID-19 questionnaire	-	Sedentary time doubled during lockdown (240 ± 240 to 480 ± 300 min day^−1^) Biggest increase in specific behaviours was electronic devices (+52.4 min day^−1^)
Gornicka et al. [51]	2381 participants No overall demographics reported	Poland	Social distancing and lockdown/homestay requirements	Canadian Health Measures Survey	-	49.1% of participants ↑ screen time 35.9% screen time ≥8 h day^−1^ on weekdays—dropping to 11.5% on weekends
Janssen et al. [52]	3241 participants 46.2 ± 15.3 years	Scotland	Social distancing and lockdown/homestay requirements	IPAQ on three occasions to track changes in sedentary behaviour	-	Sitting time ↑ 396.9 ± 188.0 min day^−1^ pre-COVID to 427.4 ± 210.9 min day^−1^ during COVID
Lopez-Bueno et al. [53]	2741 participants 34.2 ± 13.0 years	Spain	Social distancing, quarantine and lockdown/homestay requirements	Physical activity vital sign questionnaire	-	2.3% of respondents reported spending >2 h day^−1^ using screens
Luciano et al. [54]	1470 participants (394 of which assessed longitudinally) 23 ± 2 years	Italy	Social distancing and lockdown/homestay requirements	IPAQ-Short Form with additional questions added	-	Sitting time per day ↑ from 8 h day^−1^ pre-COVID to 10 h day^−1^ circa-COVID
Mon-Lopez et al. [55]	120 participants 39.6 ± 13.6 years	Spain	Social distancing and lockdown/homestay requirements	IPAQ-Short Form	-	Screen time ↑ 403.0 ± 203.4 min day^−1^ to 615.6 ± 331.6 min day^−1^
Richardson et al. [56]	117 participants 75.0 ± 4.0 years	United Kingdom	Social distancing and lockdown/homestay requirements	IPAQ-E	-	Sitting time ↑ from 426.0 ± 27.0 min day^−1^ pre-COVID to 490.0 ± 25.0 min day^−1^ during COVID
Rodrìguez-Larrad et al. [57]	13,754 university students 22.8 ± 5.3 years	Spain	Lockdown/homestay requirements	Combination of IPAQ and modified SB questions	-	Sedentary time ↑ by 52.7% from 357 ± 178 min day^−1^ (pre) to 545 ± 200 min day^−1^ (follow-up) Screen time ↑ 71.9% (217 min day^−1^–373 min day^−1^)
Rolland et al. [58]	11,391 participants 47.5 ± 17.3 years	France	Lockdown/homestay requirements	Newly developed unvalidated questionnaire	-	64.6% of people reported ↑ screen time Predictive factors included: being female (OR: 1.31) under 29 years, being single (OR: 1.15) and being employed.
Romero-Blanco et al. [59]	213 participants 20.5 ± 4.6 years	Spain	Lockdown/homestay requirements	IPAQ-Short Form	-	Sitting time ↑ 141.8 (95%CI: 71.9–141.8) min day^−1^ to 525.4 ± 194.6 min day^−1^ Normal/underweight participants sig. increased sitting time compared to overweight/obese participants Smokers sitting time did not sig. change during lockdown
Sañudo et al. [60]	20 adults 22.6 ± 3.4 years	Spain	Quarantine	Smart phone data and IPAQ-Short Form	-	Sitting time ↑ from 6.4 h day^−1^ to 9.7 h day^−1^
Savage et al. [61]	214 participants No overall demographics reported	United Kingdom	Social distancing and lockdown/homestay requirements	Exercise vital sign (EVS) questionnaireWarwick-Edinburgh Mental Well-Being Scale Perceived Stress Scale	Mental health	Sedentary time ↑ by 20 h week^−1^ during COVID restrictions Change in sedentary time was positively associated with perceived stress but not overall well-being
Stieger et al. [62]	286 participants 31.0 ± 14.5 years	Austria	Social distancing and lockdown/homestay requirements	Adapted survey questions to assess total screen time	Well-Being	↑ screen time associated with a poorer sense of well-being
Alomari et al. [63]	1844 participants 33.7 ± 1.3 years	Jordan	Social distancing and school closures	Newly developed unvalidated questionnaire	-	72.3% of participants ↑ TV time 82.7% of participants ↑ in technology usage 81.9% of participants ↑ social media
Chawla et al. [64]	231 participants No overall demographics available	India	Social distancing and lockdown/homestay requirements	IPAQ and sitting focused questions (for SB measure)	Quality of Life	33.3% reported spending ≥6 h day^−1^ screen time ≥ 6 h day^−1^ screen time associated with ↓ psychological and social well-being
Hussain and Ashkanani [65]	415 participants 38.5 ± 12.7 years	Kuwait	Lockdown/homestay requirements	Adapted questionnaires	-	% of people watching >6 h day^−1^ increased by 27.5%
Ismail et al. [66]	1012 participants No overall demographics available	United Arab Emirates	Social distancing and quarantine measures	IPAQ-Short Form with a screen time question added	-	Number of people using screen time >5 h day^−1^ for work ↑ 15.6% >5 h day^−1^ screen time for leisure-time ↑ 23.7%
Ismail et al. [67]	2970 participants No overall demographics available	Multinational	Social distancing, quarantines and lockdown/homestay requirements	IPAQ-Short Form with a screen time question added	-	Number of people using screen time >5 h day^−1^ for work ↑ 15.6% >5 h day^−1^ screen time for leisure-time ↑ 22.9%
Qi et al. [68]	645 participants 31.8 ± 8.6 years	China	Social distancing and lockdown/homestay requirements	IPAQ-Short Form and the SF-8 to assess health related quality of life	HRQoL	Sedentary time ↑ 0.4 h day^−1^ to 5.8 ± 4.6 h day^−1^ Significant negative correlation between sedentary time and perceived physical health (r^2^ = −0.10, *p* < 0.05)
Qin et al. [69]	12,107 participants No overall demographics reported	China	Lockdown/homestay requirements	IPAQ-Short Form and the positive and negative affect schedule (PANAS)	-	261.3 ± 189.8 min day^−1^ screen time
Rahman et al. [70]	2028 participants 25.9 ± 8.1 years	Bangladesh	Lockdown/homestay requirements	IPAQ-Short Form	-	20.9% of participants >8 h day^−1^ in sedentary behaviours
Wang et al. [71]	2289 participants 27.8 ± 12.0 years	China	Social distancing and lockdown/homestay requirements	New questionnaire–but good detail of measures throughout	Quality of Life Score	Average sitting time 7.4 ± 3.4 h day^−1^ SB negatively correlated to QoL (r^2^ = −0.05, *p* < 0.01)
Yang et al. [72]	10,082 participants 19.8 ± 2.3 years	China	Social distancing and school closures	IPAQ-Short Form	-	Sedentary time ↑ from 4.0 to 4.5 h day^−1^
Yilmaz et al. [73]	1120 participants 33.0 ± 11.0 years	Turkey	Social distancing and quarantine measures	New questionnaire—but good detail of measures throughout	-	Sitting time was 5.4 ± 2.6 h day^−1^
Zheng et al. [74]	631 participants 21.1 ± 2.9 years	Hong Kong	Quarantine, closure of schools and work at home orders	IPAQ Sedentary Behaviour Questionnaire (SBQ)	-	Daily SB during COVID 9.4 ± 3.0 h day^−1^ compared to 7.8 ± 3.2 h day^−1^ pre-COVID
Barkley et al. [75]	398 participants No overall demographics provided	United States	Social distancing and lockdown/homestay requirements	IPAQ	-	All university staff members reported ↑ sedentary time—average of +467 min week^−1^ Average sedentary time during COVID 481.0 ± 207.0 min day^−1^
McDowell et al. [76]	2303 participants No overall demographics provided	United States	Social distancing and lockdown/homestay requirements	Online questionnaire—limited details available	-	Mean sedentary time 533.0 ± 208.5 min day^−1^ People who began working from home, or lost their jobs, were most likely to ↑ sedentary time
Meyer et al. [77]	5036 participants No overall descriptives available	United States	Social distancing, quarantine, and lockdown/homestay requirements	IPAQ-Short Form and adapted COVID specific survey questions	-	42.6% (95% CI: 41.2–44.0%) of participants sat for >8 h day^−1^
Stephan et al. [78]	2230 participants 46.7 ± 17.8 years	United States	Social distancing and lockdown / homestay requirements	Recall questionnaires to assess sedentary behaviour	-	Time spent sedentary ↑ by 40 min day^−1^ to 7.3 ± 3.8 h day^−1^
Zajacova et al. [79]	4319 participants No overall demographics reported	Canada	Social distancing and lockdown/homestay requirements	Canadian Perspectives Survey Series 1 (CPSS-COVID)	-	66% increased TV viewing time
Browne et al. [80]	35 participants 65.6 ± 3.8 years	Brazil	Social distancing and lockdown/homestay requirements	GT3X accelerometer	-	Sedentary time ↑ 29.8 min day^−1^ to 682.6 (95%CI: 657.3–707.9) mins day^−1^ SB pattern more negative (more bouts ≥10 and 30 min, broken up less often)
Malta et al. [81]	45,161 participants No overall demographics provided	Brazil	Social distancing and lockdown/homestay requirements	Internally validated questionnaire	-	Time spent using computers/tablets ↑ 1.5 ± 0.1 h day^−1^ to 5.3 ± 0.1 h day^−1^ Time spent watching TV ↑ 1.5 ± 0.1 h day^−1^ to 3.3 ± 0.1 h day^−1^
Werneck et al. [82]	6881 participants with depression–35,143 participants with depression No overall demographics reported	Brazil	Social distancing and quarantine measures	IPAQ Long-Form	Depression	Depressed participants had significantly higher % engaging in >4 h day^−1^ TV viewing (39.6% vs. 37.4%)
Reyes-Olavarria et al. [83]	700 participants No overall demographics available	Chile	Social distancing	Recall questions to assess sedentary time	-	54.4% of participants reported spending ≥6 h day^−1^ sedentary
Asiamah et al. [84]	621 participants No overall demographics reported	Ghana	Social distancing	Newly developed questionnaire which they piloted and validated	Mental health	19.3% of participants ↑ sedentary time by ≥6 h day^−1^ Sedentary time negatively correlated with mental health
Werneck et al. [85]	38,353 participants No overall demographics reported	Brazil	Social distancing and quarantine measures	New questionnaire–but good detail of measures throughout	Mental Health (Loneliness, Sadness and Anxiety)	25% spend more than 8 h day sedentary. ↑ in the clustering of SB and physical inactivity ↑ in SB was associated with all mental health measures

ADHD = Attention Deficit Hyperactivity Disorder, CI = Confidence Intervals, COVID-19 = novel coronavirus disease 2019, IPAQ = International Physical Activity Questionnaire, OR = Odds Ratio, PA = Physical Activity, SB = Sedentary Behaviour, ST = Sedentary Time, TV = Television.

**Table 3:** Sedentary time of children during the COVID-19 pandemic by country.

Country (*n* of Papers)	Participants (*n*) and Age (Years)	Sedentary Time (Mins⋅Day^−1^)
Canada (*n* = 4)	3295 participants 11.2 ± 2.5 years	170.0 ± 96.0
China (*n* = 2)	4898 participants 16.3 ± 1.3 years	363.6 ± 148.4
Germany (*n* = 1)	1174 participants No overall demographics available	194.5 ± 141.3
Italy (*n* = 2)	112 participants 12.7 ± 2.0 years	320.0 ± 144.0
Spain (*n* = 2)	1151 participants 10.9 ± 3.3 years	330.0 ± 141.0
United States (*n* = 1)	211 participants 8.7 ± 2.6 years	480.0 ± 123.0

All values presented as mean ± standard deviation.

**Table 4:** Sedentary time of adults and older adults during the COVID-19 pandemic by country.

Country (*n* = Number of Papers)	Participants (*n*) and Age (Years)	Sedentary Time (Mins⋅Day^−1^)
Brazil (*n* = 3)	45,233 participants 56.9 ± 3.9 years	529.5 ± 20.4
Canada (*n* = 1)	351 participants 39.0 ± 5.0 years	546.0 ± 78.0
China (*n* = 4)	25,754 participants 25.1 ± 6.7 years	377.5 ± 212.5
Germany (*n* = 1)	14 participants (elite kayakers) 22.9 ± 1.4 years	729.0 ± 21.0
Italy (*n* = 2)	2900 participants 23.0 ± 2.0 years	540.0 ± 300.0
Spain (*n* = 6)	20,738 participants 30.8 ± 4.3 years	538.0 ± 258.1
Turkey (*n* = 1)	1120 participants 33.0 ± 11.0 years	324.0 ± 156.0
United Kingdom (*n* = 3)	3358 participants 60.6 ± 8.0 years	458.7 ± 118.0
United States (*n* = 3)	5031 participants 46.7 ± 17.8 years	484.0 ± 214.5

All values presented as mean ± standard deviation.

## Data Availability

The data that support the findings of this study are available from the corresponding author upon reasonable request.

## Supplementary Materials

The following are available online https://www.mdpi.com/article/10.3390/ijerph182111286/s1. Search Terms, Table S1: Risk of Bias Table—Child studies, Table S2: Risk of bias and quality assessment for all studies included involving adults and older adults.

## References

[1] Saunders T.J., Chaput J.P., Tremblay M.S. (2014). Sedentary behaviour as an emerging risk factor for cardiometabolic diseases in children and youth. Can. J. Diabetes.

[2] Väistö J., Haapala E., Viitasalo A., Schnurr T., Kilpeläinen T., Karjalainen P., Westgate K., Lakka H., Laaksonen D., Ekelund U. (2019). Longitudinal associations of physical activity and sedentary time with cardiometabolic risk factors in children. Scand. J. Med. Sci. Sports.

[3] Ku P., Steptoe A., Liao Y., Hsueh M., Chen L. (2018). A cut-off of daily sedentary time and all cause mortality in adults: A meta-regression analysis involving more than one million participants. BMC Med..

[4] Diaz K., Howard V., Hutto B., Colabianchi N., Vena J., Safford M., Blair S., Hooker S. (2017). Patterns of Sedentary Behavior and Mortality in U.S. Middle-Aged and Older Adults: A National Cohort Study. Ann. Intern. Med..

[5] Leitzmann M.F., Jochem C., Schmid D. (2018). Sedentary Behaviour Epidemiology.

[6] Wilmot E., Edwardson C., Achana F., Davies M., Gorley T., Gray L., Khunti K., Yates T., Biddle S. (2012). Sedentary time in adults and the association with diabetes, cardiovascular disease and death: Systematic review and meta-analysis. Diabetologia.

[7] Owen N., Healy G., Matthews C., Dunstan D. (2010). Too much sitting: The population-health science of sedentary behaviour. Exerc. Sport Sci. Rev..

[8] Ekelund U., Steene-Johannessen J., Brown W., Fagerland M., Owen N., Powell K., Bauman A., Lee I. (2016). Physical activity attenuates the detrimental association of sitting time with mortality: A harmonised meta-analysis of data from more than one million men and women. Lancet.

[9] Carson V., Tremblay M., Chaput J., McGregor D., Chastin S. (2019). Compositional analyses of the associations between sedentary time, different intensities of physical activity, and cardiometaolic biomarkers among children and youth from the United States. PLoS ONE.

[10] Rideout V., Foehr U., Roberts D. Generation M2: Media in the Lives of 8- to 18-Year-Olds. https://www.kff.org/other/event/generation-m2-media-in-the-lives-of/.

[11] Leatherdale S., Ahmed R. (2011). Screen-based sedentary behaviours among a nationally representative sample of youth: Are Canadian kids couch potatoes?. Chronic Dis. Inj. Can..

[12] Steene-Johannessen J., Hansen B., Dalene K., Kolle E., Northstone K., Møller N., Grøntved A., Wedderkopp N., Kriemler S., Page A. (2020). Variations in accelerometry measured physical activity and sedentary time across Europe—Harmonized analyses of 47,497 children and adolescents. Int. J. Behav. Nutr. Phys. Act..

[13] Zhang Z., Li H., Slapsinskaite A., Zhang T., Zhang L., Gui C. (2020). Accelerometer-measured physical activity and sedentary behavior in Chinese children and adolescents: A systematic review and meta-analysis. Public Health.

[14] Dempsey P., Biddle S., Buman M., Chastin S., Ekelund U., Friedenreich C., Katzmarzyk P., Leitzmann M., Stamatakis E., van der Ploeg H. (2020). New global guidelines on sedentary behaviour and health for adults: Broadening the behavioural targets. Int. J. Behav. Nutr. Phys. Act..

[15] World Health Organisation (2020). Coronavirus Disease 2019 (COVID-19) Situation Report 51.

[16] Dunford D., Dale B., Stylianou N., Lowther E., Ahmed M., de la Torre Areanas I. Coronavirus: The World in Lockdown in Maps and Charts. https://www.bbc.com/news/world-52103747.

[17] Caputo E., Reichert F. (2020). Studies of Physical Activity and COVID-19 during the Pandemic: A Scoping Review. J. Phys. Act. Health.

[18] Meyer J., McDowell C., Lansing J., Brower C., Smith L., Tully M., Herring M. (2020). Changes in Physical Activity and Sedentary Behavior in Response to COVID-19 and Their Associations with Mental Health in 3052 US Adults. Int. J. Environ. Res. Public Health.

[19] Ugbolue U., Duclos M., Urzeala C., Berthon M., Kulik K., Bota A., Thivel D., Bagheri R., Gu Y., Baker J. (2020). An Assessment of the Novel COVISTRESS Questionnaire: COVID-19 Impact on Physical Activity, Sedentary Action and Psychological Emotion. J. Clin. Med..

[20] Kang S., Sun Y., Zhang X., Sun F., Wang B., Zhu W. (2020). Is Physical Activity Associated with Mental Health among Chinese Adolescents during Isolation in COVID-19 Pandemic?. J. Epidemiol. Glob. Health.

[21] Lu C., Chi X., Liang K., Chen S., Huang L., Guo T., Jiao C., Yu Q., Veronese N., Soares F. (2020). Moving More and Sitting Less as Healthy Lifestyle Behaviors are Protective Factors for Insomnia, Depression, and Anxiety among Adolescents during the COVID-19 Pandemic. Psychol. Res. Behav. Manag..

[22] Moher D., Shamseer L., Clarke M., Ghersi D., Liberati A., Petticrew M., Shekelle P., Stewart L.A., Group P.-P. (2015). Preferred Reporting Items for Systematic Review and Meta-Analysis Protocols (PRISMA-P) 2015 Statement. Syst. Rev..

[23] Shamseer L., Moher D., Clarke M., Ghersi D., Liberati A., Petticrew M., Shekelle P., Stewart L.A., Group P.-P. (2015). Preferred reporting items for systematic review and meta-analysis protocols (PRISMA-P) 2015: Elaboration and explanation. BMJ.

[24] Higgins J., Green S. Handbook for Systematic Reviews of Interventions Version 5.1.0. https://handbook-5-1.cochrane.org/.

[25] Janssen I., Clarke A., Carson V., Chaput J., Giangregorio L., Kho M., Poitras V., Ross R., Saunders T., Ross-White A. (2020). A systematic review of compositional data analysis studies examining associations between sleep, sedentary behaviour, and physical activity with health outcomes in adults. Appl. Physiol. Nutr. Metab. = Physiol. Appl. Nutr. Metab..

[26] Poitras V., Gray C., Janssen X., Aubert S., Carson V., Faulkner G., Goldfield G., Reilly J., Sampson M., Tremblay M. (2017). Systematic review of the relationships between sedentary behaviour and health indicators in the early years (0–4 years). BMC Public Health.

[27] Garcia J., Lawrence S., Brazendale K., Leahy N., Fukuda D. (2020). Brief report: The impact of the COVID-19 pandemic on health behaviors in adolescents with Autism Spectrum Disorder. Disabil. Health J..

[28] Sciberras E., Patel P., Stokes M., Coghill D., Middeldorp C., Bellgrove M., Becker S., Efron D., Stringaris A., Faraone S. (2020). Physical Health, Media Use, and Mental Health in Children and Adolescents with ADHD during the COVID-19 Pandemic in Australia. J. Atten. Disord..

[29] Pietrobelli A., Pecoraro L., Ferruzzi A., Heo M., Faith M., Zoller T., Antoniazzi F., Piacentini G., Fearnbach S., Heymsfield S. (2020). Effects of COVID-19 Lockdown on Lifestyle Behaviors in Children with Obesity Living in Verona, Italy: A Longitudinal Study. Obesity.

[30] López-Bueno R., López-Sánchez G., Casajús J., Calatayu J., Gil-Salmerón A., Grabovac I., Tully M., Smith L. (2020). Health-Related Behaviors among School-Aged Children and Adolescents during the Spanish COVID-19 Confinement. Front. Pediatr..

[31] Medrano M., Cadenas-Sanchez C., Oses M., Arenaza L., Amasene M., Labayen I. (2021). Changes in lifestyle behaviours during the COVID-19 confinement in Spanish children: A longitudinal analysis from the MUGI project. Pediatr. Obes..

[32] Palladino F., Merolla E., Solimeno M., de Leva M., Lenta S., Di Mita O., Bonadies A., Striano P., Tipo V., Varone A. (2020). Is COVID-19 lockdown related to an increase of accesses for seizures in the emergency department? An observational analysis of a paediatric cohort in the Southern Italy. Neurol. Sci. Off. J. Ital. Neurol. Soc. Ital. Soc. Clin. Neurophysiol..

[33] Dutta K., Mukherjee R., Sen D., Sahu S. (2020). Effect of COVID-19 lockdown on sleep behaviour and screen exposure time: An observational study among Indian school children. Biol. Rhythm Res..

[34] Eyimaya A., Irmak Y. (2020). Relationship between parenting practices and children’s screen time during the COVID-19 Pandemic in Turkey. J. Pediatr. Nurs..

[35] Munasinghe S., Sperandei S., Freebairn L., Conroy E., Jani H., Marjanovic S., Page A. (2020). The Impact of Physical Distancing Policies during the COVID-19 Pandemic on Health and Well-Being among Australian Adolescents. J. Adolesc. Health Off. Publ. Soc. Adolesc. Med..

[36] Carroll N., Sadowski A., Laila A., Hruska V., Nixon M., Ma D., Haines J., On Behalf Of The Guelph Family Health Study (2020). The Impact of COVID-19 on Health Behavior, Stress, Financial and Food Security among Middle to High Income Canadian Families with Young Children. Nutrients.

[37] McCormack G., Doyle-Baker P., Petersen J., Ghoneim D. (2020). Parent anxiety and perceptions of their child’s physical activity and sedentary behaviour during the COVID-19 pandemic in Canada. Prev. Med. Rep..

[38] Schmidt S., Anedda B., Burchartz A., Eichsteller A., Kolb S., Nigg C., Niessner C., Oriwol D., Worth A., Woll A. (2020). Physical activity and screen time of children and adolescents before and during the COVID-19 lockdown in Germany: A natural experiment. Sci. Rep..

[39] Dunton G., Do B., Wang S. (2020). Early effects of the COVID-19 pandemic on physical activity and sedentary behavior in children living in the U.S. BMC Public Health.

[40] Mitra R., Moore S., Gillespie M., Faulkner G., Vanderloo L., Chulak-Bozzer T., Rhodes R., Brussoni M., Tremblay M. (2020). Healthy movement behaviours in children and youth during the COVID-19 pandemic: Exploring the role of the neighbourhood environment. Health Place.

[41] Moore S.A., Faulkner G., Rhodes R.E., Brussoni M., Chulak-Bozzer T., Ferguson L.J., Mitra R., O’Reilly N., Spence J.C., Vanderloo L.M. (2020). Impact of the COVID-19 virus outbreak on movement and play behaviours of Canadian children and youth: A national survey. Int. J. Behav. Nutr. Phys. Act..

[42] Francisco R., Pedro M., Delvecchio E., Espada J., Morales A., Mazzeschi C., Orgilés M. (2020). Psychological Symptoms and Behavioral Changes in Children and Adolescents during the Early Phase of COVID-19 Quarantine in Three European Countries. Front. Psychiatry.

[43] Zinner C., Matzka M., Leppich R., Kounev S., Holmberg H., Sperlich B. (2020). The Impact of the German Strategy for Containment of Coronavirus SARS-CoV-2 on Training Characteristics, Physical Activity and Sleep of Highly Trained Kayakers and Canoeists: A Retrospective Observational Study. Front. Sports Act. Living.

[44] Rezende D., Pinto A., Goessler K., Nicoletti C., Sieczkowska S., Meireles K., Esteves G., Genario R., Oliveira Júnior G., Santo M. (2021). Influence of Adherence to Social Distancing Due to the COVID-19 Pandemic on Physical Activity Level in Post-bariatric Patients. Obes. Surg..

[45] Biviá-Roig G., La-Rosa V., Gómez-Tébar M., Serrano-Raya L., Amer-Cuenca J., Caruso S., Commodari E., Barrasa-Shaw A., Lisón J. (2020). Analysis of the Impact of the Confinement Resulting from COVID-19 on the Lifestyle and Psychological Wellbeing of Spanish Pregnant Women: An Internet-Based Cross-Sectional Survey. Int. J. Environ. Res. Public Health.

[46] Werneck A., Silva D., Malta D., Souza-Júnior P., Azevedo L., Barros M., Szwarcwald C. (2021). Physical inactivity and elevated TV-viewing reported changes during the COVID-19 pandemic are associated with mental health: A survey with 43,995 Brazilian adults. J. Psychosom. Res..

[47] Castañeda-Babarro A., Arbillaga-Etxarri A., Gutiérrez-Santamaría B., Coca A. (2020). Physical Activity Change during COVID-19 Confinement. Int. J. Environ. Res. Public Health.

[48] Cheval B., Sivaramakrishnan H., Maltagliati S., Fessler L., Forestier C., Sarrazin P., Orsholits D., Chalabaev A., Sander D., Ntoumanis N. (2021). Relationships between changes in self-reported physical activity, sedentary behaviour and health during the coronavirus (COVID-19) pandemic in France and Switzerland. J. Sports Sci..

[49] Colivicchi F., Di Fusco S., Magnanti M., Cipriani M., Imperoli G. (2020). The Impact of the Coronavirus Disease-2019 Pandemic and Italian Lockdown Measures on Clinical Presentation and Management of Acute Heart Failure. J. Card. Fail..

[50] Gallè F., Sabella E., Ferracuti S., De Giglio O., Caggiano G., Protano C., Valeriani F., Parisi E., Valerio G., Liguori G. (2020). Sedentary Behaviors and Physical Activity of Italian Undergraduate Students during Lockdown at the Time of CoViD-19 Pandemic. Int. J. Environ. Res. Public Health.

[51] Górnicka M., Drywień M., Zielinska M., Hamułka J. (2020). Dietary and Lifestyle Changes during COVID-19 and the Subsequent Lockdowns among Polish Adults: A Cross-Sectional Online Survey PLifeCOVID-19 Study. Nutrients.

[52] Janssen X., Fleming L., Kirk A., Rollins L., Young D., Grealy M., MacDonald B., Flowers P., Williams L. (2020). Changes in Physical Activity, Sitting and Sleep across the COVID-19 National Lockdown Period in Scotland. Int. J. Environ. Res. Public Health.

[53] López-Bueno R., Calatayud J., Casaña J., Casajús J., Smith L., Tully M., Andersen L., López-Sánchez G. (2020). COVID-19 Confinement and Health Risk Behaviors in Spain. Front. Psychol..

[54] Luciano F., Cenacchi V., Vegro V., Pavei G. (2021). COVID-19 lockdown: Physical activity, sedentary behaviour and sleep in Italian medicine students. Eur. J. Sport Sci..

[55] Mon-López D., Bernardez-Vilaboa R., Fernandez-Balbuena A., Sillero-Quintana M. (2020). The Influence of COVID-19 Isolation on Physical Activity Habits and Its Relationship with Convergence Insufficiency. Int. J. Environ. Res. Public Health.

[56] Richardson D., Duncan M., Clarke N., Myers T., Tallis J. (2021). The influence of COVID-19 measures in the United Kingdom on physical activity levels, perceived physical function and mood in older adults: A survey-based observational study. J. Sports Sci..

[57] Rodríguez-Larrad A., Mañas A., Labayen I., González-Gross M., Espin A., Aznar S., Serrano-Sánchez J.A., Vera-Garcia F.J., González-Lamuño D., Ara I. (2021). Impact of COVID-19 Confinement on Physical Activity and Sedentary Behaviour in Spanish University Students: Role of Gender. Int. J. Environ. Res. Public Health.

[58] Rolland B., Haesebaert F., Zante E., Benyamina A., Haesebaert J., Franck N. (2020). Global Changes and Factors of Increase in Caloric/Salty Food Intake, Screen Use, and Substance Use during the Early COVID-19 Containment Phase in the General Population in France: Survey Study. JMIR Public Health Surveill..

[59] Romero-Blanco C., Rodríguez-Almagro J., Onieva-Zafra M., Parra-Fernández M., Prado-Laguna M., Hernández-Martínez A. (2020). Physical Activity and Sedentary Lifestyle in University Students: Changes during Confinement Due to the COVID-19 Pandemic. Int. J. Environ. Res. Public Health.

[60] Sanudo B., Fennell C., Sanchez-Oliver A. (2020). Objectively-assessed physical activity, sedentary behaviour, smart phone use, and sleep patterns pre-and during-COVID-19 quarantine in young adults from Spain. Sustainability.

[61] Savage M., James R., Magistro D., Donaldson D., Healy L., Nevill M., Hennis P. (2020). Mental health and movement behaviours during the COVID-19 pandemic in UK university students: Prospective cohort study. Ment. Health Phys. Act..

[62] Stieger S., Lewetz D., Swami V. (2021). Emotional Well-Being under Conditions of Lockdown: An Experience Sampling Study in Austria during the COVID-19 Pandemic. J. Happiness Stud..

[63] Alomari M., Khabour O., Alzoubi K. (2020). Changes in Physical Activity and Sedentary Behavior Amid Confinement: The BKSQ-COVID-19 Project. Risk Manag. Healthc. Policy.

[64] Chawla B., Chawla S., Singh H., Jain R., Arora I. (2020). Is coronavirus lockdown taking a toll on mental health of medical students? A study using WHOQOL-BREF questionnaire. J. Fam. Med. Prim. Care.

[65] Husain W., Ashkanani F. (2020). Does COVID-19 change dietary habits and lifestyle behaviours in Kuwait: A community-based cross-sectional study. Environ. Health Prev. Med..

[66] Ismail C., Osaili T., Mohamad M., Al Marzouqi A., Jarrar A., Abu Jamous D., Magriplis E., Ali H., Al Sabbah H., Hasan H. (2020). Eating Habits and Lifestyle during COVID-19 Lockdown in the United Arab Emirates: A Cross-Sectional Study. Nutrients.

[67] Ismail C., Osaili T., Mohamad M., Al Marzouqi A., Jarrar A., Zampelas A., Habib-Mourad C., Jamous O., Ali H., Al Sabbah H. (2021). Assessment of eating habits and lifestyle during the coronavirus 2019 pandemic in the Middle East and North Africa region: A cross-sectional study. Br. J. Nutr..

[68] Qi M., Li P., Moyle W., Weeks B., Jones C. (2020). Physical Activity, Health-Related Quality of Life, and Stress among the Chinese Adult Population during the COVID-19 Pandemic. Int. J. Environ. Res. Public Health.

[69] Qin F., Song Y., Nassis G., Zhao L., Dong Y., Zhao C., Feng Y., Zhao J. (2020). Physical Activity, Screen Time, and Emotional Well-Being during the 2019 Novel Coronavirus Outbreak in China. Int. J. Environ. Res. Public Health.

[70] Rahman M., Islam M., Bishwas M., Moonajilin M., Gozal D. (2020). Physical inactivity and sedentary behaviors in the Bangladeshi population during the COVID-19 pandemic: An online cross-sectional survey. Heliyon.

[71] Wang X., Lei S., Le S., Yang Y., Zhang B., Yao W., Gao Z., Cheng S. (2020). Bidirectional Influence of the COVID-19 Pandemic Lockdowns on Health Behaviors and Quality of Life among Chinese Adults. Int. J. Environ. Res. Public Health.

[72] Yang S., Guo B., Ao L., Yang C., Zhang L., Zhou J., Jia P. (2020). Obesity and activity patterns before and during COVID-19 lockdown among youths in China. Clin. Obes..

[73] Yılmaz S., Eskici G. (2021). Evaluation of emotional (depression) and behavioural (nutritional, physical activity and sleep) status of Turkish adults during the COVID-19 pandemic period. Public Health Nutr..

[74] Zheng C., Huang W., Sheridan S., Sit C., Chen X., Wong S. (2020). COVID-19 Pandemic Brings a Sedentary Lifestyle in Young Adults: A Cross-Sectional and Longitudinal Study. Int. J. Environ. Res. Public Health.

[75] Barkley J., Lepp A., Glickman E., Farnell G., Beiting J., Wiet R., Dowdell B. (2020). The Acute Effects of the COVID-19 Pandemic on Physical Activity and Sedentary Behavior in University Students and Employees. Int. J. Exerc. Sci..

[76] McDowell C., Herring M., Lansing J., Brower C., Meyer J. (2020). Working From Home and Job Loss Due to the COVID-19 Pandemic Are Associated with Greater Time in Sedentary Behaviors. Front. Public Health.

[77] Meyer J., Herring M., McDowell C., Lansing J., Brower C., Schuch F., Smith L., Tully M., Martin J., Caswell S. (2020). Joint prevalence of physical activity and sitting time during COVID-19 among US adults in April 2020. Prev. Med. Rep..

[78] Stephan Y., Terracciano A., Luchetti M., Aschwanden D., Lee J., Sesker A., Strickhouser J., Sutin A. (2021). Physical Activity and Sedentary Behaviour during COVID-19: Trajectory and Moderation by Personality. Soc. Psychol. Personal. Sci..

[79] Zajacova A., Jehn A., Stackhouse M., Denice P., Ramos H. (2020). Changes in health behaviours during early COVID-19 and socio-demographic disparities: A cross-sectional analysis. Can. J. Public Health = Rev. Can. Sante Publique.

[80] Browne R., Macêdo G., Cabral L., Oliveira G., Vivas A., Fontes E., Elsangedy H., Costa E. (2020). Initial impact of the COVID-19 pandemic on physical activity and sedentary behavior in hypertensive older adults: An accelerometer-based analysis. Exp. Gerontol..

[81] Malta D., Szwarcwald C., Barros M., Gomes C., Machado Í., Souza Júnior P., Romero D., Lima M., Damacena G., Pina M. (2020). The COVID-19 Pandemic and changes in adult Brazilian lifestyles: A cross-sectional study, 2020. Epidemiol. Serv. Saude Rev. Sist. Unico Saude Bras..

[82] Werneck A.O., da Silva D.R., Malta D.C., de Souza P.R.B., Azevedo L.O., Barros M.B.D., Szwarcwald C.L. (2020). Lifestyle behaviors changes during the COVID-19 pandemic quarantine among 6881 Brazilian adults with depression and 35,143 without depression. Cienc. Saude Coletiva.

[83] Reyes-Olavarría D., Latorre-Román P., Guzmán-Guzmán I., Jerez-Mayorga D., Caamaño-Navarrete F., Delgado-Floody P. (2020). Positive and Negative Changes in Food Habits, Physical Activity Patterns, and Weight Status during COVID-19 Confinement: Associated Factors in the Chilean Population. Int. J. Environ. Res. Public Health.

[84] Asiamah N., Opuni F., Mends-Brew E., Mensah S., Mensah H., Quansah F. (2021). Short-Term Changes in Behaviors Resulting from COVID-19-Related Social Isolation and Their Influences on Mental Health in Ghana. Community Ment. Health J..

[85] Werneck A., Silva D., Malta D., Souza-Júnior P., Azevedo L., Barros M., Szwarcwald C. (2021). Changes in the clustering of unhealthy movement behaviors during the COVID-19 quarantine and the association with mental health indicators among Brazilian adults. Transl. Behav. Med..

[86] Sá C., Pombo A., Luz C., Rodrigues L., Cordovil R. (2020). COVID-19 Social Isolation in Brazil: Effects on the Physical Activity Routine of Families with Children. Rev. Paul. Pediatr. Orgao Of. Soc. Pediatr. Sao Paulo.

[87] Gray C., Gibbons R., Larouche R., Sandseter E., Bienenstock A., Brussoni M., Chabot G., Herrington S., Janssen I., Pickett W. (2015). What Is the Relationship between Outdoor Time and Physical Activity, Sedentary Behaviour, and Physical Fitness in Children? A Systematic Review. Int. J. Environ. Res. Public Health.

[88] Love R., Adams J., Atkin A., van Sluijs E. (2019). Socioeconomic and ethnic differences in children’s vigorous intensity physical activity: A cross-sectional analysis of the UK Millennium Cohort Study. BMJ Open.

[89] Akpinar A. (2017). Urban green spaces for children: A cross-sectional study of associations with distance, physical activity, screen time, general health, and overweight status. Urban For. Urban Green..

[90] Saunders T., Tremblay M., Mathieu M., Henderson M., O’Loughlin J., Tremblay A., Chaput J. (2013). Associations of sedentary behavior, sedentary bouts and breaks in sedentary time with cardiometabolic risk in children with a family history of obesity. PLoS ONE.

[91] Egan C., Webster C., Beets M., Weaver R., Russ L., Michael D., Nesbitt D., Orendorff K. (2019). Sedentary time and Behaviour during School: A systematic Review and Meta-Analysis. Am. J. Health Educ..

[92] Abbott R., Straker L., Mathiassen S. (2013). Patterning of children’s sedentary time at and away from school. Obesity (Silver Spring Md.).

[93] Lubans D., Hesketh K., Cliff D., Barnett L., Salmon J., Dollman J., Morgan P., Hills A., Hardy L. (2011). A systematic review of the validity and reliability of sedentary behaviour measures used with children and adolescents. Obes. Rev. Off. J. Int. Assoc. Study Obes..

[94] Ottevaere C., Huybrechts I., De Bourdeaudhuij I., Sjostrom M., Ruiz J.R., Ortega F.B., Hagstromer M., Widhalm K., Molnar D., Moreno L.A. (2011). Comparison of the IPAQ-A and ActiGraph in relation to VO2 max among European adolescents: The HELENA study. Sci. Med. Sport.

[95] Tremblay M.S., Leblanc A.G., Janssen I., Kho M.E., Hicks A., Murumets K., Colley R.C., Duggan M. (2011). Canadian sedentary behaviour guidelines for children and youth. Appl. Physiol. Nutr. Metab..

[96] Colley R., Garriguet D., Janssen I., Wong S., Saunders T., Carson V., Tremblay M. (2013). The association between accelerometer-measured patterns of sedentary time and health risk in children and youth: Results from the Canadian Health Measures Survey. BMC Public Health.

[97] Ekelund U., Luan J., Sherar L., Esliger D., Griew P., Cooper A. (2012). Moderate to vigorous physical activity and sedentary time and cardiometabolic risk factors in children and adolescents. JAMA.

[98] Carson V., Tremblay M., Chaput J., Chastin S. (2016). Associations between Sleep Duration, Sedentary Time, Physical Activity, and Health Indicators among Canadian Children and Youth Using Compositional Analyses. Appl. Physiol. Nutr. Metab..

[99] Prince S., Roberts K., Melvin A., Butler G., Thompson W. (2020). Gender and education differences in sedentary behaviour in Canada: An analysis of national cross-sectional surveys. BMC Public Health.

[100] Kallio J., Hakonen H., Syväoja H., Kulmala J., Kankaanpää A., Ekelund U., Tammelin T. (2020). Changes in physical activity and sedentary time during adolescence: Gender differences during weekdays and weekend days. Scand. J. Med. Sci. Sports.

[101] Hidding L., Chinapaw M., van Poppel M., Mokkink L., Altenburg T. (2018). An Updated Systematic Review of Childhood Physical Activity Questionnaires. Sports Med..

[102] Hallal P., Anderson L., Bull F., Guthold R., Haskell W., Ekelund U. (2012). Global physical activity levels: Surveillance progress. pitfalls, and prospects. Lancet.

[103] WHO (2020). WHO Guidelines on Physical Activity and Sedentary Behaviour.

[104] Arigo D., Pasko K., Mogle J. (2020). Daily relations between social perceptions and physical activity among college women. Psychol. Sport Exerc..

[105] Balish S., Deaner R., Rainham D., Blanchard C. (2016). Sex Differences in Sport Remain When Accounting for Countries’ Gender Inequality. Cross-Cult. Res..

[106] Copeland J.L., Eslinger D.W. (2009). Accelerometer assessment of physical activity in active, healthy older adults. J. Ageing Phys. Act..

[107] Burki T. (2020). China’s successful control of COVID-19. Lancet. Infect. Dis..

[108] Schmid D., Ricci C., Leitzmann M.F. (2015). Associations of objectively assessed physical activity and sedentary time with all-cause mortality in US adults: The NHANES study. PLoS ONE.

[109] Hamer M., Coombs N., Stamatakis E. (2014). Associations between objectively assessed and self-reported sedentary time with mental health in adults: An analysis of data from the Health Survey for England. BMJ Open.

[110] Mattioli A.V., Sciomer S., Cocchi C., Maffei S., Gallina S. (2020). Quarantine during COVID-19 outbreak: Changes in diet and physical activity increase the risk of cardiovascular disease. Nutr. Metab. Cardiovasc. Dis..

[111] Gradidge P.J.L., Kruger H.S. (2020). Physical activity, diet and quality of life during mandatory (COVID-19) quarantine following repatriation. SAGE Open Med. Case Rep..

