# Supplementary material for: Impact of the COVID-19 Pandemic on Sedentary Time and Behaviour in Children and Adults: A Systematic Review and Meta-Analysis
Source: Int J Environ Res Public Health. 2021 Oct 27;18(21):11286. doi: 10.3390/ijerph182111286 (PMC8583678; doi:10.3390/ijerph182111286)
Supplement: Supplementary file 1 [file ijerph-18-11286-s001.zip › ijerph-1367666-supplementary.pdf]

Supplementary Matierals

Impact of the COVID-19 Pandemic on Sedentary Time and Behaviour in Children and Adults: A Systematic Review and Meta-Analysis

Search Terms:

"Sedentary behav*" OR "Sedentary time" OR "sedent*" OR "physical inactiv*" OR "physical in-activ*" OR "screen time" OR "screen use" OR "screen view*" OR "sitting" OR "prolonged sitting" OR "sedentary postur*"

AND

"Coronavirus" OR "corona-virus" OR "COVID-19" OR "Covid19" OR "SARS-CoV*" OR "SARSCov2" OR "SARSCOV2" OR "2019 coronavirus" OR "2019 corona virus" OR "novel corona virus" OR "2019-nCoV" OR "nCoV*" OR "ncov*"

Table 1. Risk of Bias Table – Child studies.

Reference	Detection Bias		Attrition Bias		Selection Bias		Performance Bias		Selective Reporting Bias		Other Sources of Bias		GRADE Score
	Rating	Rationale for rating	Rating	Rationale for rating	Rating	Rationale for rating	Rating	Rationale for rating	Rating	Rationale for rating	Rating	Rationale for rating	
Kang et al. [20]	Mod	Subjectively measured via questionnaires	Low	92.1% of questionnaires had valid data	Low	Randomised sample recruited from 49 schools	Mod.	Used the IPAQ-Short Form	Low	Outcomes reported in specified ways	High	Also considered mental health and PA levels	Mod.
Lu et al. [21]	Mod.	Subjectively measured via questionnaires	Low	91.6% of eligible children participated	Low	Sampled from ten local schools	Mod.	Used IPAQ-Short Form	Low	Outcomes reported in specified ways	Low	Also considered participants mental health and demographics	High
Garcia et al. [27]	Mod	Subjectively measured using recall questionnaires	Not Clear	Only included participants reported	Mod.	All recruited from the same private school	Mod.	Subjectively assessed using recall questionnaires	Low	Outcomes reported in specified ways	Mod.	Longitudinal study design	Mod.
Sciberras et al [28]	Mod.	Subjectively measured from questionnaires	Not Clear	Only completed questionnaires reported	Mod.	Recruited from an ongoing longitudinal study	Mod.	Assessed using the CoRonavIruS Health Impact Survey (CRISIS)	Low	Outcomes reported in specified ways	High	No other covariates considered	Mod.
Pietrobelli et al. [29]	Low	Interview questions – answered by parents	Low	82% of cohort completed both arms	Mod.	All recruited from an ongoing longitudinal study	Mod.	Used two different means of interview techniques	Low	Outcomes reported in specified ways	Mod.	Also assessed food habits and PA changes during lockdown	High

Lopez-Bueno et al. [30]	Mod	Subjectively measured via questionnaire	Not Clear	Only completed questionnaires completed	Low	Randomised sample from social media platforms	Mod.	Used the physical activity vital sign (PAVS) questionnaire	Low	Outcomes reported in specified ways	High	No other covariates considered in analyses	Low
Medrano et al. [31]	Mod.	Subjectively measured via questionnaires	High	Only 42.1% of participants followed longitudinally	Mod.	Recruited from on-going longitudinal study	Mod.	Used the youth PA (YPA) questionnaire	Low	Outcomes reported in specified ways	High	No other covariates considered	Low
Palladino et al. [32]	Mod.	Sample of children with siezures in an Italian Hospital	Not Clear	Only included participants reported	Mod.	All recruited from one hospital in Italy	Mod.	Used questionnaires to assess total screen time	Low	Outcomes reported in specified ways	Mod.	Correlations run between total sedentary time and seizure likelihood	High`
Dutta et al. [33]	Mod.	Subjectively assessed using questionnaires	High	Only 56.5% of questionnaires had valid data	Low	Randomised sample form social media platforms	Mod.	Recall questionnaires used to estimate screen time	Low	Outcomes reported in specified ways	High	No other covariates considered	Mod.
Eyimaya & Irmak [34]	Mod.	Subjectively measured but completed by parents	Mod.	~70% of all eligible participants completed the survey	Low	Distributed to local schools in the area	Low	Used parenting practice scale (PPS)	Low	Outcomes reported in specified ways	High	No other covariates considered	Mod.
Munasinghe et al. [35]	Mod	Subjectively measured using questionnaires	Not Clear	Only participants in the prospective cohort study reported	Mod.	Sample taken from pre-existing cohort study	Mod.	Used the PACE + Adolescent Physical Activity Measures	Low	Outcomes reported in specified ways	High	No other covariates considered	Mod.
Carroll et al. [36]	Mod.	Part of pre-existing	Not Clear	Only included participants reported	Mod	Sample taken from existing	Mod.	Parental recall of childs PA and SB	Low	Outcomes reportedin	Mod.	SES and diet also included	Mod.

		longitudinal study				longitudinal study				specified ways			
McCormack et al. [37]	Mod.	Subjectively measured via questionnaires	Not Clear	Only completed questionnaires reported	Low	Random sample of adults	Mod.	Parental recall of child's PA and sedentary time	Low	Outcomes reported in specified ways	High	No other covariates considered in analyses	Mod.
Schmidt et al. [38]	Mod.	Subjectively measured from questionnaires	Not Clear	Only completed questionnaires reported	Mod.	Recruited form an ongoing cohort study (Motorik-Modul)	Mod.	Assessed using the MoMo PA questionnaire	Low	Outcomes reported in specified ways	Mod.	Also considered changes in habitual PA and sports activity changes	Mod.
Dunton et al. [39]	Mod.	Subjectively assessed using questionnaires	Not Clear	Only completed questionnaire data reported	High	Convenience sample	Mod.	Parental recall of the previous days PA and SB	Low	Outcomes reported in specified ways	Low	Also considered sex, age, ethnicity, race, employment status of parents and SES	Mod.
Mitra et al. [40]	Mod.	Subjectively measured using questionnaires	Low	>99% of all participants had full data sets	Mod.	Convenience sampling using mass emails	Mod.	Used online questionnaires – limited details on specifics	Low	Outcomes reported in specified ways	Mod.	Controlled for age of child and living environment (urban V.S. rural)	High
Moore et al. [41]	Mod.	Subjectively measured using questionnaires	Low	97.9% of all completed questionnaires contained valid data	Not Clear	No specific details on how survey was distributed	Mod.	Used the particiPACTION survey	Low	Outcomes reported in specified ways	High	No other covariates considered	High

Francisco et al. [42]	Mod	Subjectively measured using questionnaires	Not Clear	Only included participants reported	Low	Randomised sample recruited from social media platforms	Mod.	Used parental recall to estimate child sedentary time	Low	Outcomes reported in specified ways	High	No other covariates considered	Mod.

Mod. = Moderate, PA = Physical Activity, SB = Sedentary Behaviour, SES = Socioeconomic Status, IPAQ = International Physical Activity Questionnaire.

Table S2. Risk of bias and quality assessment for all studies included involving adults and older adults

Reference	Detection Bias		Attrition Bias		Selection Bias		Performance Bias		Selective Reporting Bias		Other Sources of Bias		GRADE Score
	Rating	Rationale for rating	Rating	Rationale for rating	Rating	Rationale for rating	Rating	Rationale for rating	Rating	Rationale for rating	Rating	Rationale for rating	
Meyer et al. [18]	Mod.	Subjectively measured using questionnaires	Low	94.1% of all questionnaires contained valid data	Mod.	Convenience sampling using mass emailing	Mod.	Used online questionnaires – no details on specific ones used	Low	Outcomes reported in specified ways	Mod.	Controlled for lockdown restrictions and mental health outcomes	High
Carrol et al. [36]	Mod.	Part of pre-existing longitudinal study	Not Clear	Only included participants reported	Mod	Sample taken from existing longitudinal study	Mod.	Recall of seven-day PA	Low	Outcomes reportedin specified ways	Mod.	SES and diet also included	Mod.
Zinner et al. [43]	Mod.	Subjectively measured from questionnaires	Not Clear	Only included participants reported	Mod.	Wrist worn HR monitoring from which MET's calculated	Mod.	HR monitoring	Low	Outcomes reported in specified ways	Mod	Changes in training regimes, intensity and physical fitness	Mod.
Rezende et al. [44]	Low	Objective (accelerometers) and subjective (questionnaires) used	Not Clear	Only included participants reported	Mod	All recruited from the same hospital in Sao Paulo	Low	Used GT3X accelerometers and questionnaires	Low	Outcomes reported in specified ways	High	No other covariates considered	Mod.
Bivia-Roig et al [45]	Mod	Subjectively measured by questionnaires	High	67.7% had full data sets	Mod.	All recruited from maternity wards	Low	Used adapted questionnaires for SB and EuroQol-5D for mental health	Low	Outcomes reported in specified ways	Low	HRQoL, Dietary changes and adherence to guidelines considered	Mod.
Werneck et al. [87]	Mod.	Subjectively measured	Low	84.9% had full data sets	Low	Chained sampling procedure used	Mod.	Used new questionnaire – but good detail	Low	Outcomes reported in	Low	Correlates run for sex, age, region,	High

		from adapted questionnaire						of measures throughout		specified ways		education, employment & SES	
Castaneda-Babarro et al. [25]	Mod.	Subjectively measured using questionnaires	Low	91.3% of participants had full data sets	Low	Randomised sample of adults recruited	Low	Used IPAQ Short version	Low	Outcomes reported in specified ways	Mod.	Age, height, weight, sex, and employment status also recorded	High
Cheval et al. [27]	Mod.	Subjectively assessed using questionnaires	High	40.3% of participants replied to both questionnaires	Low	Randomised sample from social media platforms	Low	Used IPAQ and sitting focused questions (for SB measure)	Low	Outcomes reported in specified ways	Low	Also considered mental & physical health, age, BMI.	Mod.
Colivvicchi et al. [49]	Mod.	Subjectivley assessed using telephone interviews	Not Clear	Only included participants reported	Low	Randomised sampling methodology used	Mod.	Used recall techniques via telephone interviews	Low	Outcomes reported in specified ways	High	No other covariates considered in analyses	Mod.
Gallé et al. [50]	Mod.	Subjectively measured using questionnaires	High	0.9% of all students emailed completed the survey	Low	Recruited from three different universities	Low	Used the IPAQ and the adult sedentary behaviour questionnaire (ASBQ)	Low	Outcomes reported in specified ways	High	No other covariates considered in analyses	Low
Gornicka et al. [51]	Mod.	Subjectively measured using questionnaires	Low	92.4% of all questionnaires were retained for analyses	Low	Randomised sample recruited from social media platforms	Mod.	Used the PLifeCOVID-19 questionnaire	Low	Outcomes reported in specified ways	High	No other covariates considered in analyses	Mod.
Janssen et al. [52]	Mod.	Subjectively measured via questionnaire	Not Clear	Only completed questionnaires reported	Low	Randomised sample recruited from	Mod.	Used the IPAQ on three occasions to track changes in SB	Low	Outcomes reported in specified ways	High	No other covariates considered in analyses	Mod.

						social media platforms						
Lopez-Beuno et al. [53]	Mod.	Subjectively measured via questionnaire	Not Clear	Only completed questionnaires reported	Low	Randomised sample from social media platforms	Mod.	Adapted pre-existing questionnaires	Low	Outcomes reported in specified ways	Mod.	Also considered education level, underlying health condition, and exposure to COVID-19	High
Luciano et al. [54]	Mod.	Subjectively measured via questionnaires	Low	>95% of all participants had full data sets	Mod.	6th year medical students at an Italian University	Mod.	IPAQ-Short Form with additional questions added	Low	Outcomes reported in specified ways	High	No other covariates considered in analyses	Mod.
Mon-Lopez et al. [55]	Mod.	Subjectively measured using questionnaires	Not Clear	Only completed questionnaires data reported	Mod.	Snowballing technique (of email chains)	Mod.	Used the IPAQ-Short Form	Low	Outcomes reported in specified ways	High	No other covariates considered	Low
Richardson et al. [56]	Mod.	Subjectively measured using questionnaires	Not Clear	Only included participants reported	Mod.	Randomised sample from online platforms	Mod.	Used the IPAQ-E	Low	Outcomes reported in specified ways	High	No other covariates considered	Mod
Rodrìguez-Larrad et al. [57]	Mod.	Subjectively measured from adapted questionnaire	High	500,000 surveys sent – 13,754 responses (~2.8%)	Low	Survey distributed to 16 universities	Mod.	Combination of IPAQ and modified SB questions	Low	Outcomes reported in specified ways	High	No other covariates considered	Low
Romero-Blanco et al. [58]	Mod.	Subjectively measured from questionnaires	Not Clear	Only completed responses reported	Low	Randomised sample from university students	Mod.	Used IPAQ-Short Form	Low	Outcomes reported in specified ways	Mod.	Correlates run for health behaviours, diet and anxiety/depression scores	Mod.

Rolland et al. [59]	Mod.	Subjectively measured from adapted questionnaire	Low	Not reported – only complete survey numbers	Low	Randomised sample from social media platforms	High	Used a newly developed unvalidated questionnaire	Low	Outcomes reported in specified ways	Low	Correlates run for age, sex, marital status, employment, education, history of problems with mental health and addiction, SES and diet	Mod.
Sañudo et al. [60]	Mod.	Objectively assessed using smart phone data and subjectively using questionnaires	High	Only 20/57 (35%) completed both arms of the study	Mod.	Randomised sample from student email requests	Mod.	Objectively assessed using smart phone data and used the IPAQ-Short Form questionnaire	Low	Outcomes reported in specified ways	High	No other covariates considered	Mod.
Savage et al. [61]	Mod.	Subjectively measured using questionnaires	High	Only 22.6% of initial participants completed all four questionnaires	Mod.	Randomised sample from student email requests	Mod.	Exercise vital sign (EVS) questionnaire Warwick-Edinburgh Mental Well-Being Scale Perceived Stress Scale	Low	Outcomes reported in specified ways	Mod.	Mental health and changes in PA also considered	High
Steiger et al. [62]	Mod.	Adapted survey questions to assess total screen time	Not Clear	Not reported – only complete survey numbers	Low	Randomised sample recruited from social media platforms	High	Used a newly developed unvalidated questionnaire	Low	Outcomes reported in specified ways	High	No other covariates considered	Mod.
Alomari et al. [63]	High	Subjectively measured from new questionnaire	Not Clear	Not reported – only complete survey numbers	Low	Randomised sample recruited from	High	Used a newly developed unvalidated questionnaire	Low	Outcomes reported in specified ways	Low	Assessed strength of associations against BMI, education & income	Mod.

						social media platforms							
Chawla et al. [64]	Mod.	Subjectively measured using questionnaires	Mod.	77% of population responded to survey	Mod.	Sample of medical students at a Indian University	Mod.	Used the WHO QOL-BREF questionnaire	Low	Outcomes reported in specified ways	High	No other covariates considered	Mod.
Hussain & Ashkanani [65]	Mod.	Subjectively measured using questionnaires	Mod.	81.4% of respondents provided reliable data	Low	Randomly recruited via online distribution	Low	Used adapted questionnaires	Low	Outcomes reported in specified ways	High	No other covariates considered in analyses	Mod.
Ismail et al. [66]	Mod.	Subjectively measured via questionnaire	Not Clear	Only completed questionnaires reported	Low	Randomised sample recruited from social media platforms	Mod.	IPAQ-Short Form with a screen time question added	Low	Outcomes reported in specified ways	Mod.	Also considered dietary changes, Stress, Irritability and Sleep	High
Ismail et al. [67]	Mod.	Subjectively measured via questionnaire	Not Clear	Only completed questionnaires reported	Low	Randomised sample recruited from social media platforms	Mod.	IPAQ-Short Form with a screen time question added	Low	Outcomes reported in specified ways	Mod.	Also considered dietary changes, Stress, Irritability and Sleep	High
Qi et al. [68]	Mod.	Subjectively measured using a questionnaire	Not Clear	Only completed questionnaires reported	Mod.	Randomised sample from text invites through phone network	Mod.	Used the IPAQ-Short Form and the SF-8 to assess health related quality of life	Low	Outcomes reported in specified ways	Mod.	Also measured health related quality of life, SES, urban/rural living and marital status	High
Qin et al. [69]	Mod.	Subjectively measured from questionnaire	Not Clear	Only completed questionnaires reported	Mod.	Randomised sample from phone network and	Mod.	Used the IPAQ-Short Form and the positive and negative	Low	Outcomes reported in specified ways	High	No other covariates considered	Mod.

						social media platforms		affect schedule (PANAS)					
Rahman et al. [70]	Mod.	Subjectively measured from questionnaire	Not Clear	Only completed questionnaires reported	Mod.	Random sample from social media platforms	Mod.	IPAQ-Short Form	Low	Outcomes reported in specified ways	High	No other covariates assessed	Mod.
Wang et al. [71]	Mod.	Subjectively measured from questionnaire	Mod.	Response rate of 76.3%	Not Clear	No clear details about how the questionnaire was distributed	Mod.	Sedentary time assessed using the IPAQ-Short Form	Low	Outcomes reported in specified ways	Mod.	Also considered QoL, dietary behaviours and PA	High
Yang et al. [72]	Mod.	Subjectively measured from questionnaires	Not Clear	Only completed questionnaires reported	Low	Randomised recruitment from social media platforms	Mod.	Subjectively measured using IPAQ long-form	Low	Outcomes reported in specified ways	High	No other covariates considered	Mod.
Yilmaz et al. [73]	Mod.	Subjectively measured using questionnaires	Not Clear	Only completed questionnaires reported	Low	Recruited through emails or social media platforms	High	No specific details on questionnaires used	Low	Outcomes reported in specified ways	Mod.	Also considered mental health, diet and knowledge of COVID-19	High
Zheng et al. [74]	Mod.	Subjectively measured from questionnaires	Not Clear	Only completed surveys reported	Mod.	Measured using the sedentary behaviour questionnaire (SBQ) and IPAQ	Mod.	Used validated, existing questionnaire	Low	Outcomes reported in specified ways	High	No other covariates considered in analyses	Mod.

Barkley et al. [75]	Mod.	Subjectively measured from questionnaires	Low	96.3% of eligible participants included	Low	Randomised sample recruited from a mid-western university	Low	Used the IPAQ	Low	Outcomes reported in specified ways	High	No other covariates considered in the analyses	Mod.
McDowell et al. [76]	Mod.	Subjectively measured via questionnaires	Not Clear	Only completed questionnaires reported	Mod.	Convenience email sampling	Mod.	Online questionnaire – limited	Low	Outcomes reported in specified ways	Mod.	Changes in employment status and demographics added to linear model	Mod.
Meyer et al. [77]	Mod.	Subjectively assessed using questionnaires	Mod.	80.3% of all questionnaires contained valid data	Low	Random sampling 'snowballing' method used	Mod.	Used IPAQ-Short Form and adapted COVID specific survey questions	Low	Outcomes reported in specified ways	High	No other covariates considered	Mod.
Stephan et al. [78]	Mod.	Recall question-naires	Not Clear	Only completed questionnaires reported	Mod.	Convenience email sampling	Mod.	Online questionnaire – limited	Low	Outcomes reported in specified ways	High	No other covariates considered in analyses	Mod.
Zajacova et al. [79]	Mod.	Subjectively measured from questionnaires	Low	93.3% of all completed questionnaires had full data sets	Mod.	Used public use data from the Canadian Perspectives Survey Series	Mod.	Used adapted questionnaire	Low	Outcomes reported in specified ways	Mod.	Covaried for age, sex, SES, diet and substance use in analyses	High
Browne et al. [80]	Low	Objectively measured using accelerometers	Low	87.5% of participants had valid data	Mod.	Recruited from an ongoing study	Low	Used the GT3X accelerometer	Low	Outcomes reported in specified ways	High	No other covariates considered in analyses	Mod.

Malta et al. [81]	Mod.	Subjectively measured via questionnaires	Low	95.7% of all data had full data sets	Mod.	Chain sampling procedure	Mod.	Internally validated questionnaire	Low	Outcomes reported in specified ways	High	No other covariates considered in analyses	Mod.
Werneck et al. [82]	Mod	Subjectively measured from adapted questionnaire	Not Clear	Only completed questionnaires reported	Low	Chained sampling procedure used	Mod.	Used new questionnaire – but good detail of measures throughout	Low	Outcomes reported in specified ways	High	No other covariates considered in analyses	Mod.
Reyes-Olavarria et al. [83]	Mod.	Subjectively measured from questionnaire	Not Clear	Only completed questionnaires reported	High	Convenience sampling method	High.	Used recall questions to assess ST	Low	Outcomes reported in specified ways	High	No other covariates considered	Low
Asiamah et al. [84]	Mod.	Subjectively assessed using questionnaires	Not Clear	Not reported – only complete survey numbers	Low	Randomised sample recruited from social media platforms	Mod.	Used a newly developed questionnaire which they piloted and validated	Low	Outcomes reported in specified ways	Mod.	Mental health, PA and changes in unhealthy behaviours (smoking, drug and alcohol use) considered	High
Werneck et al. [46]	Mod.	Subjectively measured from adapted questionnaire	Low	97.4% had full data sets	Low	Chained sampling procedure used	Mod.	Used new questionnaire – but good detail of measures throughout	Low	Outcomes reported in specified ways	Low	Covaried for age, sex, education, job status, ethnicity, alcohol and tobacco use, family health, and quarantine adherence	High

Mod. = Moderate, PA = Physical Activity, SB = Sedentary Behaviour, SES = Socioeconomic Status, IPAQ = International Physical Activity Questionnaire
